# Aldehyde dehydrogenase 2 activation and coevolution of its εPKC-mediated phosphorylation sites

**DOI:** 10.1186/s12929-016-0312-x

**Published:** 2017-01-05

**Authors:** Aishwarya Nene, Che-Hong Chen, Marie-Hélène Disatnik, Leslie Cruz, Daria Mochly-Rosen

**Affiliations:** Department of Chemical and Systems Biology, Stanford University, School of Medicine, Stanford, CA 94305-5174 USA

**Keywords:** ALDH2, Aldehyde dehydrogenase 2, εPKC, Phosphorylation, Coevolution, 4HNE

## Abstract

**Background:**

Mitochondrial aldehyde dehydrogenase 2 (ALDH2) is a key enzyme for the metabolism of many toxic aldehydes such as acetaldehyde, derived from alcohol drinking, and 4HNE, an oxidative stress-derived lipid peroxidation aldehyde. Post-translational enhancement of ALDH2 activity can be achieved by serine/threonine phosphorylation by epsilon protein kinase C (εPKC). Elevated ALDH2 is beneficial in reducing injury following myocardial infarction, stroke and other oxidative stress and aldehyde toxicity-related diseases. We have previously identified three εPKC phosphorylation sites, threonine 185 (T185), serine 279 (S279) and threonine 412 (T412), on ALDH2. Here we further characterized the role and contribution of each phosphorylation site to the enhancement of enzymatic activity by εPKC.

**Methods:**

Each individual phosphorylation site was mutated to a negatively charged amino acid, glutamate, to mimic a phosphorylation, or to a non-phosphorylatable amino acid, alanine. ALDH2 enzyme activities and protection against 4HNE inactivation were measured in the presence or absence of εPKC phosphorylation in vitro. Coevolution of ALDH2 and its εPKC phosphorylation sites was delineated by multiple sequence alignments among a diverse range of species and within the ALDH multigene family.

**Results:**

We identified S279 as a critical εPKC phosphorylation site in the activation of ALDH2. The critical catalytic site, cysteine 302 (C302) of ALDH2 is susceptible to adduct formation by reactive aldehyde, 4HNE, which readily renders the enzyme inactive. We show that phosphomimetic mutations of T185E, S279E and T412E confer protection of ALDH2 against 4HNE-induced inactivation, indicating that phosphorylation on these three sites by εPKC likely also protects the enzyme against reactive aldehydes. Finally, we demonstrate that the three ALDH2 phosphorylation sites co-evolved with εPKC over a wide range of species. Alignment of 18 human ALDH isozymes, indicates that T185 and S279 are unique ALDH2, εPKC specific phosphorylation sites, while T412 is found in other ALDH isozymes. We further identified three highly conserved serine/threonine residues (T384, T433 and S471) in all 18 ALDH isozymes that may play an important phosphorylation-mediated regulatory role in this important family of detoxifying enzymes.

**Conclusion:**

εPKC phosphorylation and its coevolution with ALDH2 play an important role in the regulation and protection of ALDH2 enzyme activity.

**Electronic supplementary material:**

The online version of this article (doi:10.1186/s12929-016-0312-x) contains supplementary material, which is available to authorized users.

## Background

The mitochondrial aldehyde dehydrogenase 2, ALDH2, is known for its role in ethanol metabolism, mediating the rate-limiting step of metabolizing acetaldehyde to acetic acid [1]. However, this enzyme is also critical for oxidation of fatty acid-derived aldehydes, such as 4-hydrox-2-nonenal (4HNE) to non-electrophilic and unreactive acids, 4-hydroxy-2-enoic acid (4HNA) [2, 3]. Therefore, ALDH2 plays a critical physiological role both in the removal acetaldehyde derived from alcohol drinking and the detoxification of lipid peroxidation by-products, 4HNE, under oxidative stress.

The functional ALDH2 is a homotetramer [4]. In human, a single point mutation in ALDH2 (E487K) greatly reduces the enzyme’s activity [5–7]. This over-dominant mutation, designated as ALDH2*2, is found in nearly 40% of East Asian populations, or approximately 560 million of the world population [8–10]. ALDH2*2 mutation leads to high levels of acetaldehydes accumulation in the blood after ethanol consumption and causes the well-known Asian Alcohol Flushing Syndrome [9, 11]. Because of the accumulation of acetaldehyde, a known Group 1 carcinogen [12], the inactive variant of ALDH2*2 is associated with a much higher incidence of upper aerodigestive track cancers as well as gastric, colorectal, lung, and hepatocellular cancersc; a meta-analysis suggests up to 80 fold higher incidence in heterozygotes who drink more than 9 alcoholic beverages per week [9, 13–15].

The α,β-unsaturated reactive 4HNE is well-known for its genotoxicity and cytotoxicity, causing DNA damage and proteins inactivation [16–18]. 4HNE is reactive and readily forms Michael’s adducts on the nucleophilic amino acids, cysteine, histidine and lysine [19, 20]. Many protein targets of 4HNE have been identified, including both serum and cellular components, such as albumin and histones, and cytoprotective proteins, critical protein quality control, such as HSP70, and the 20S proteasome [21, 22]. Since 4HNE is a product of lipid peroxidation and the mitochondrial respiratory electron transport chain is the major source of ROS, it is likely that many of the mitochondrial proteins are susceptible to 4HNE modification. Indeed, a notably large proportion of the 4HNE modified proteins that have been identified reside in the mitochondria [22]. These include critical proteins in respiratory chain and energy metabolism, such as aconitase, ATP synthase, many dehydrogenases in the Krebs cycle and, importantly, ALDH2 itself [23, 24]. 4HNE is a substrate of ALDH2, but is also a potent inhibitor of ALDH2, since it can readily inactivate this enzyme by adducting to critical cysteine residue in the catalytic active site, cysteine 302 (Cys 302) [24, 25]. Inactivation of ALDH2 by its own substrate, 4HNE, therefore could lead to further accumulation of 4HNE, which has been observed in many pathological conditions including neurodegenerative, ischemic and inflammatory diseases [26–29].

Enhancing the catalytic activity of ALDH2 and/or protecting ALDH2 enzyme activity from 4HNE-induced inactivation has recently emerged as new strategy for the development of therapeutics [26, 27]. Our lab has identified small molecules activators of ALDH2 (e.g., Alda-1) that increase the catalytic activity of the enzyme directly and also protect ALDH2 from 4HNE substrate-induced inactivation [27]. X-ray co-crystal structure of Alda-1 and ALDH2 showed that Alda-1 is bound at the substrate tunnel of ALDH2, close to cysteine 302, thus likely shielding and preventing the thiol-group of this amino acid from interacting with 4HNE [30]. In the absence of Alda-1, we showed that ALDH2 was rapidly inactivated by 4HNE within minutes. Whereas in the presence of Alda-1, ALDH2 remained catalytically active for an extended period of time [31].

**Fig. 1:**
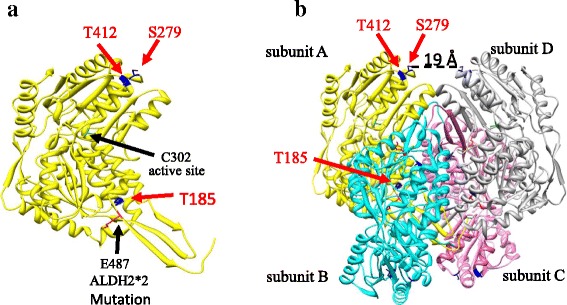
Structure of ALDH2 enzyme. **a** ALDH2 monomer displaying the three phosphorylation sites identified by LC-MS-MS: Thr185, Ser279, and Thr412 (*blue*). Also highlighted are the catalytic Cys302 (green) and the site of the ALDH2*2 or Asian mutation: Glu487 (*red*). **b** Tetramer of an active ALDH2 enzyme form. Thr185, Ser279 and Thr412 are marked in subunits A as in (**a**). The distance of between the two Ser279s on subunits *A* and *D* is also indicated

Another way to enhance ALDH2 activity is by post-translational phosphorylation of the enzyme. We previously found that activation of epsilon protein kinase C (εPKC) at the mitochondria increases ALDH2 activity in the heart by ~40%, thus protecting the heart from ischemic injury [31, 32]; phosphorylation of ALDH2 by εPKC increases metabolism of toxic aldehydes, including 4HNE. However, the molecular basis for phosphorylation-induced activation of the enzyme is not known. Using liquid chromatography and mass spectrometry analysis we identified previously three possible εPKC-mediated phosphorylation sites on ALDH2 (Chen et al., 2008 supporting online material and Fig. 1). These are serine 279 (S279), which lies at N-terminal end of helix that immediately precedes the catalytic residue Cys 302, threonine 185 (T185), which lies in the loop between end of the first helix in the enzyme, and threonine 412 (T412), which lies at the N-terminus of an α-helix [31]. However, the importance of these phosphorylation sites for the enzymatic activity and the role (if any) of phosphorylation at these sites in protecting ALDH2 against 4HNE inactivation are not known.

Using site-directed mutagenesis of the three possible εPKC phosphorylation sites, we set out to determine their role in enzyme activity, phosphorylation, folding, and resistance to 4HNE inactivation. We also explored the conservation of these sites with εPKC in evolution, as a means to demonstrate their importance in regulating ALDH2.

## Methods

### Enzyme activity assay for aldehyde dehydrogenase

Enzymatic activity of ALDH2 was determined spectrophotometrically, using purified recombinant protein to measure the reductive reaction of NAD^+^ to NADH at λ340 nm. All the assays were carried out in a 96-well plate in triplicates at 30 °C in 50 mM sodium pyrophosphate buffer, pH = 8.8, 2.5 mM NAD+ and 10 mM acetaldehyde as the substrate, as described [31]. ALDH2 activities were expressed as μmole NADH/min/μg protein from the linear range of the assay. The amount of mutant ALDH2 recombinant protein in each sample was determined by Bradford assays and quantitative western blots, using commercial bovine serum albumin and a highly purified wild type ALDH2 as a standard. Where indicated, 4HNE (50 μM) was added at the start of the kinetic assays immediately following the addition of acetaldehyde. All kinetic assays were measured for sixty minutes.

### Site-directed mutagenesis and purification of human recombinant enzymes of ALDH2 wild type, ALDH2*2 and T185, S279, T412 phosphorylation site mutants

Human recombinant ALDH2 wild type and ALDH2*2 mutant enzymes were expressed in bacteria as previously described [31]. The PKC-mediated three phosphorylation sites identified previously by LC/MS/MS, Thr185, Ser279 and Thr412 were mutated to glutamic acid, to mimic phosphorylation [33] or to alanine, as a control. For sited-directed mutagenesis, primers were designed and mutations were introduced by AccuPrime™ Pfx DNA polymerase kit for cloning and mutagenesis according to manufacture protocol (Life Technologies; catalog number 12344–024). ALDH2 wild type clone was used as the PCR template. Primer sets used for each site-directed mutagenesis are as following: T185A (Forward: GCCCAGCCTTGGCAGCTGGAAACGTGGTT; Reverse: AACCACGTTTCCAGCTGCCAAGGCTGGGC), T185E (Forward: GCTGGGCCCAGCCTTGGCAGAGGGAAACGTGGTTGTG; Reverse: CACAACCACGTTTCCCTCTGCCAAGGCTGGGCCCAGC), S279A (Forward: GCCCCAACATCATCATGGCAGATGCCGATATGGAT; Reverse: ATCCATATCGGCATCTGCCATGATGATGTTGGGGC), S279E (Forward: AAGAGCCCCAACATCATCATGGAGGATGCCGATATGGATTGGGC; Reverse: GCCCAATCCATATCGGCATCCTCCATGATGATGTTGGGGCTCTT), T412A (Forward: CAGATCCTGAAGTTCAAGGCCATAGAGGAGGTTGTTG; Reverse: CAACAACCTCCTCTATGGCCTTGAACTTCAGGATCTG), T412E (Forward: ATGCAGATCCTGAAGTTCAAGGAGATAGAGGAGGTTGTTGGGAGA; Reverse: TCTCCCAACAACCTCCTCTATCTCCTTGAACTTCAGGATCTGCAT). All the constructed human ALDH2 wild type and mutants were designed to express a recombinant protein with the His-tag at the N-terminus of the protein using E. coli BL21 host cells and purified by His GraviTrap nickel-affinity column (GE Healthcare Life Sciences) as described previously [31].

### Phosphorylation of ALDH2 recombinant proteins by εPKC

For in vitro kinase reaction, recombinant εPKC (100 ng, Life Technologies, Grand Island, NY, USA) and each ALDH2 protein (8 μg) were incubated in the presence of 20 mM Tris–HCl pH 7.5, 200 μM ATP, 20 mM MgCl_2_ with 0.24 mg/ml phosphatidylserine (Avanti, AL, USA), 0.04 mg/ml 1,3-s-n-dioleylglycerol (Avanti, Alabaster, AL) at 37 °C for 30 min as described in Chen et al. [31].

### Protein sequence, structural alignment and analysis

Sequences for members of the ALDH family and the ALDH2 protein from multiple species were found through the NCBI protein database (see Additional file 1). The sequence alignment of ALDH2 proteins from multiple species was determined by using the NCBI Constraint-based Multiple Protein Alignment Tool (COBALT). Structures of the different ALDH2 mutants were modeled using UCSF Chimera by running a sequence alignment to reduce the Root Mean Square Deviation. Structural analyses were carried out to determine whether the phosphomimetic mutations (T185E, S279E and T412E) affect the protein structure. Each mutation was introduced using the MOE (Molecular Operating Environment) program. Following energy minimization, the protein model was searched for areas where the mutated residue would clash with other surrounding residues using the UCSF Chimera program which searches for atoms that have a Van der Waals radius overlap of 0.6 angstroms and ignores contacts of pairs that are 2 or fewer bonds apart.

### Amino acid sequence alignment of 18 human ALDH isozymes

19 different, functional ALDH genes are known in the human genome [27]. Since ALDH18A1 showed very low degree of homology with the rest of the 18 ALDH isozyme and has no conservation of T185, S279 and T412 at the equivalent positions, it was omitted from our sequence alignment. Multiple sequence alignment was conducted using online software ClustalW (http://embnet.vital-it.ch/software/ClustalW.html) and ALDH sequences with the following GenBank Accession numbers: ALDH2 (GI: 48146099), ALDH1A1 (GI: 16306661), ALDH1A2 (GI: 119597936), ALDH1A3 (GI: 153266822), ALDH1B1 (GI: 119578656), ALDH1L1 (GI: 393195306), ALDH1L2 (GI:166198355), ALDH3A1 (GI: 206597441), ALDH3A2 (GI:73466520), ALDH3B1 (GI:125950429), ALDH3B2 (GI: 73695881), ALDH4A1 (GI: 23271000), ALDH5A1 (GI: 21708023), ALDH6A1 (GI: 119601566), ALDH7A1 (GI: 49117277), ALDH8A1 (GI: 88683005), ALDH9A1 (GI: 119611164), ALDH16A1 (GI: 223972651). For longer sequences of ALDH isozymes, both N- and C-terminal sequences were truncated and small sequence gaps were introduced to obtain the best fitted alignment against the published ALDH2 protein sequence.

## Results

The common East Asian ALDH2*2 single point mutation (E487K), which is away from the catalytic site, causes a >95% loss of activity in ALDH2 due to structural changes that affect both the dimerization of the enzyme and binding of the cofactor, NAD^+^ [34]. To determine whether phosphorylation causes a global change in ALDH2 structure, in silico analysis of structural models was carried out (Fig. 1a). Ser 279 lies on the surface of the catalytic domain, near the dimer-dimer interface, between the A/B dimer and C/D dimer, such that the residue is ~19 Å from its subunit related Ser [A subunit and D subunit] (Fig. 1b). Ser 279 lies at the N-terminal end of the helix that immediately precedes the catalytic Cys (302) and is 27 Å from Cys302. [For comparison, Glu487, which is mutated to Lys in ALDH2*2, is 17 Å from Cys302.] Thr412, located at the N-terminus of an α-helix, is only 10 Å from Ser279 on the surface of the catalytic domain, though it is further from the subunit interface. Finally, Thr185 residue is in the loop between the end of the first helix and the beginning of the second strand in the Rossmann coenzyme-binding fold [34, 35]. Thr185 is 9 Å from Glu487, the mutated amino acid in ALDH2*2. Therefore, Thr185 is adjacent to an area of the enzyme that is known to affect activity and catalysis. Although it appears buried, it is accessible to solvent if the C-terminal residues contributed by a subunit in the opposing dimer of the tetramer are displaced. Phosphorylation of Thr185 is predicted to preclude the binding of the C-terminal carboxylate through electrostatic repulsion (Fig. 1).

**Fig. 2:**
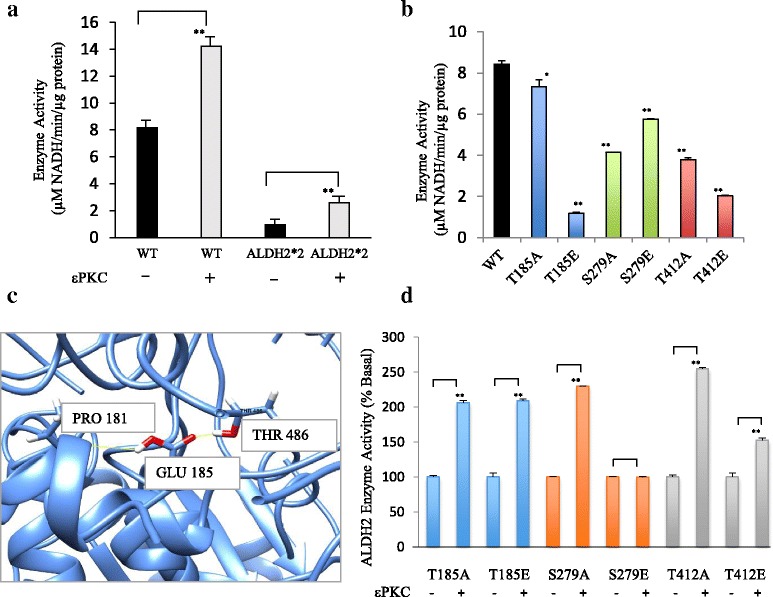
εPKC phosphorylation on wild type ALDH2, ALDH2*2 and Thr185, Ser279 and Thr412 mutant enzymes. **a** Increased activity for wild type ALDH2 and ALDH2*2 mutant enzymes by εPKC phosphorylation**.** ALDH2 wild type (WT) and ALDH2*2 mutant enzyme activities were measured in the absence or presence of εPKC. Enzyme activity was expressed in μmole NADH/min/μg recombinant protein (*n* = 3, **p < 0.001; bars represent the mean ± SD). **b** Enzymatic active of the phosphomimetic ALDH2 site-directed mutants, T185E, S279E and T412E. Enzyme activity was expressed in μmole NADH/min/μg recombinant protein (*n* = 3, *p < 0.05, **p < 0.001 *vs.* WT; bars represent the mean ± SD). **c** A structural analysis of the T185E mutation reveals that a glutamate in the position of T185 would clash with the surrounding amino acids, proline 181 and threonine 486. **d** The effect of εPKC phosphorylation on the phosphomimetic and non-phosphorylatable mutants of ALDH2. The graph displays the enzyme activity of T185, S279 and T412 mutants with or without the phosphorylation of εPKC (*n* = 3, *p < 0.05, **p < 0.001; bars represent the mean ± SD)

We have reported previously that in vitro phosphorylation of wild type ALDH2 recombinant protein increases its enzymatic activity [31]. We observed here an increase of 70% the ALDH2 activity following phosphorylation by recombinant εPKC (Fig. 2a). The effect of εPKC phosphorylation on ALDH2*2 mutant enzyme was even more pronounced, even though the ALDH2*2 mutant enzyme had a much lower basal activity due to the Glu487 substitution by Lys. As shown in Fig. 2a, we observed that the enzymatic activity of the phosphorylated ALDH2*2 is 270% of the non-phosphorylated ALDH2*2.

We set out to determine which of the phosphorylation sites contributes to εPKC-mediated activation of ALDH2 enzymatic activity. Site-directed mutagenesis was carried out for each of the putative εPKC phosphorylation sites, Thr185, Ser279 and Thr412 on ALDH2. Since phosphomimetic of an amino acid is a good estimation for the function of phosphorylation, we first mutated the three phosphorylation sites individually to a charged amino acid residue, glutamate, to mimic the function of the negatively charged phosphate group [33]. We found that all the single phosphomimetic ALDH2 mutants were less active than the wild type ALDH, especially T185E. Compared to the non-phosphorylated wild type ALDH2, the T185E, S279E and T412E had only 14%, 68%, and 24% of the wild type activity, respectively (Fig. 2b). A structural model of the T185E mutant suggests that a mutation to glutamate at position 185 will likely cause a conformational change (Fig. 2c), as the glutamate residue at that position appears to clash with the surrounding amino acids, proline 181 and threonine 486. This prediction is supported by replacing the glutamate residue to an alanine residue. When the T185 phosphorylation site was mutated to alanine to serve as a non-phosphorylatable control, the enzymatic activity of the mutant enzyme was not affected as much as compared with the T185E mutant. In this case, T185A retains 87% of the wild type ALDH2 activity (Fig. 2b). In contrast, the S279A and T412A mutants showed a loss ~50% (49% for S279A and 45% for T412A) of the wild type activity (Fig. 2b). Interestingly, among the three phosphomimetics, the S279E phosphomimetic was the only mutant that had approximately 40% higher activity relative to its S279A non-phosphorylatable mutant, suggesting that S279 is probably a true allosteric site, capable of increasing the catalytic activity of ALDH2 upon phosphorylation. Similar to T185, either alanine or glutamate substitution for T412 decreased the catalytic activity of ALDH2. However, our structural modeling did not indicate any clashes with surrounding amino acids for the T412E substitution (Fig. 2c).

Next, we determined whether further activation of the enzymatic activity could be achieved by εPKC phosphorylation of each of the single phosphomimetic or non-phosphorylatable alanine substitution mutants. We reasoned that since further activation by εPKC phosphorylation on the specific phosphomimetic or non-phosphorylatable alanine substitution was no longer possible, such experiments will help to identify the true phosphorylation site(s) contributing the enhanced ALDH2 enzyme activity by εPKC. We found that five out of six possible amino acid substitutions, T185A/E and T412A/E and S279A mutants, were significantly activated by εPKC-mediated phosphorylation, resulting in an increase of 50–150% above their basal activity (Fig. 2d). The phosphomimetic S279E mutant was clearly the only exception; it was insensitive to further activation by εPKC-mediated phosphorylation. These data are consistent with the observation above that phosphomimetic substitution, S279E, was the mutation that imparted the highest increased in ALDH2 activity without phosphorylation and that S279 phosphorylation is the critical event in εPKC-mediated activation of ALDH2.

**Fig. 3:**
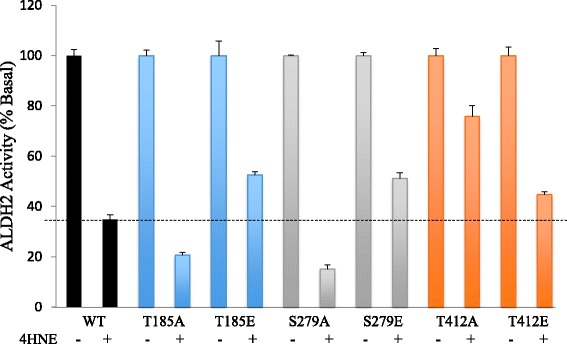
Sensitivity of the non-phosphorylatable and phosphomimetic ALDH2 mutants to 4HNE inactivation. Enzyme activity of each of the T185, S279 and T412 single phosphorylation mutant (*A* or *E*) with or without incubation with 50 μM of 4HNE. All enzyme activities are presented as a percentage of the no 4HNE treatment for each of the mutant

Because 4HNE causes a fast inactivation of ALDH2 by adduct formation with the critical catalytic Cys302 [24, 36], we also determined whether phosphorylation mimetic mutations protect the enzyme and affect the sensitivity of ALDH2 to 4HNE-induced inactivation. We showed that wild type ALDH2 enzyme activity decreases rapidly by ~65% immediately after addition of 50 μM 4HNE (Fig. 3). Compared to the wild type ALDH2, the non-phosphorylatable mutants, T185A or S279A were more sensitive to 4HNE inactivation and lost 79% and 85% of their activity, respectively. Surprisingly, the T412A mutation only lost 24% activity and was more resistant to 4HNE inactivation than the wild type. Importantly, the phosphomimetic mutations, T185E and S279E, increased the resistance to 4HNE-induced inactivation. Compared to a 65% decrease in the wild type, ALDH2 activity of T185E and S279E mutant enzymes showed only a decrease of 47% and 49%, respectively (Fig. 3). On the other hand, although the phosphomimetic T412E mutant was not as resistant to 4HNE-induced inactivation as the T412A mutant, it conferred some protection to ALDH2 after incubation with 4HNE with a 55% reduction of activity as compared to the loss 65% observed from the wild type ALDH2 (Fig. 3). The simplest explanation for these results is that phosphorylation on ALDH2 may induce a conformational change in the enzyme structure thus allosterically protect Cys302 adduction by 4HNE.

**Fig. 4:**
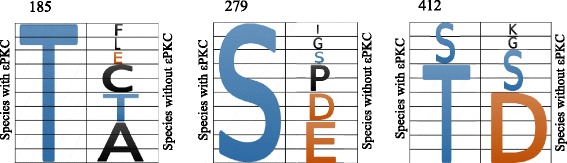
Co-evolution of εPKC and phosphorylation residues in ALDH2. Shown is the amino acid (one-letter code) at the indicated position in ALDH2 from 20 different evolutionarily diverged species. Each cell represents one species. The left column depicts the amino acids at that site for the ten species that have εPKC. The right hand column depicts the amino acids at that corresponding site (determined by alignment of the whole sequence) for the ten species that do not have εPKC. In both columns, the size of the amino acid represents the frequency of the given amino acid at that site. Residues that can be phosphorylated by εPKC, serine and threonine, are colored in blue. Residues colored in red are negative amino acids, thus mimicking the phosphorylated serine and threonine. Other amino acids are colored in black. For a list of the 20 species, their phylogenetic tree and their respective amino acid residues at corresponding T187, S279 and T412, see Additional files 1 and 2

We also used multiple sequence alignments to determine whether the three ALDH2 phosphorylation sites were conserved among species and co-evolved with εPKC. We reasoned that if εPKC-mediated phosphorylation of ALDH2 is critical for the regulation of ALDH2 activity, the critical phosphorylation sites should co-evolve with εPKC. We aligned multiple sequences of ALDH2 from a wide range of eukaryotic species that express εPKC, and compared ALDH2 sequence conservation with species that do not express this protein kinase (Additional files 1 and 2). Focusing on T185, S279 and T412-equivalent phosphorylation positions in ALDH2, we compared the conservation of the regions corresponding to the phosphorylated site in 10 species that express εPKC and 10 species that lack εPKC (Fig. 4, Additional files 1 and 2). Remarkably, in the 10 species that express εPKC, either a serine or a threonine was invariably found at the three putative phosphorylation sites in ALDH2 (Fig. 4, left columns). In contrast, in the 10 species that lack εPKC, conservation of phosphorylatable amino acids, T185, S279, T412 was minimal (Fig. 4, right columns).

It is expected that in the absence of a kinase, if a phosphorylation site is important for an enzymatic activity or biological function, that position will be substituted for by a negative amino acid (glutamate or aspartate) to mimic phosphorylation [37]. We found that, for ALDH2 T185, among the 10 species that did not express εPKC only 1 of the 10 species had a negative amino acid. For both S279 and T412, out of the 10 species that lacked εPKC, half had a negative amino acid in the place of the phosphorylation site. These data are consistent with the idea that evolutionary conservation of a negatively charged amino acid for a phosphorylatable serine/threonine in that position indicates a functionally important residue for activity. In addition, we also found that in several species that did not express εPKC, serine or threonine was still conserved. 2 of the 10 species that did not have εPKC retained threonine at position T185, 1 of 10 species retained serine at position S279 and 2 of 10 species had a serine substitution at the equivalent for T412 position. These data suggest that in the absence of εPKC, another serine/threonine protein kinase may phosphorylate ALDH2 in these species.

**Fig. 5:**
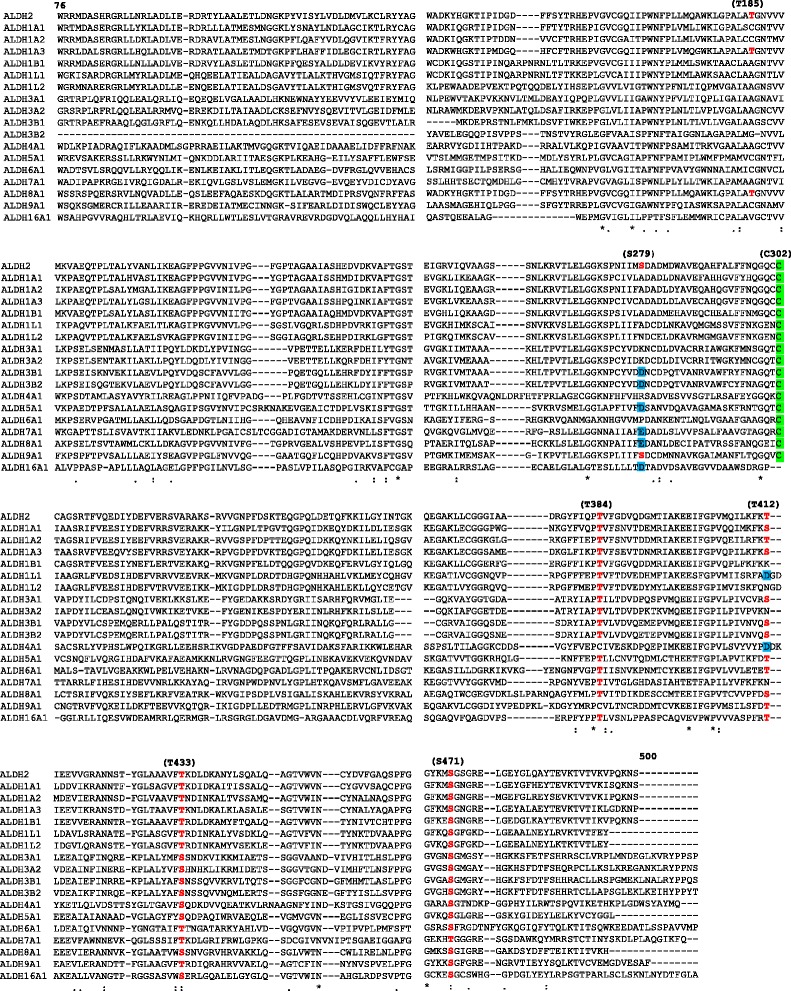
Alignment of amino acid sequences of the 18 human ALDH isozymes. Amino acid sequences of 18 human ALDH isozymes were aligned based on their sequence homology. For longer ALDH isozymes, both N- and C-terminal sequences were truncated to obtain the best fitted alignment against the ALDH2 protein sequence from its amino acid residues 76 to 500 as marked (without the 17 a.a. N-terminal mitochondria targeting sequence). Serine and threonine at positions T187, S279, T384, T412, T433 and S471 the conserved are denoted in red letters. The negatively charged amino acids, D and E, are in blue. The conserved catalytic site, Cysteine 302 (C302) residues, are marked in green. For GenBank accession numbers of all ALDH isozymes, see Methods

We also aligned and compared amino acid sequences of all 19 identified and functional ALDH isozymes within the human genome and determined how the positions equivalent to T185, S279 and T412 are conserved among the human ALDH supergene family (Fig. 5). We reasoned that such a comparison will reveal whether other ALDH isozymes may also be regulated by phosphorylation (perhaps even by εPKC-mediated phosphorylation) in a similar fashion. Because ALDH18A1 showed very low degree of homology with the rest of the 18 ALDH isozyme and no conservation of an equivalent to T185, S279 and T412 was found, it was omitted from this comparison. Figure 5 depicts the best alignment of the remaining 18 human ALDH isozymes. We found that the equivalents of either T185 or S279 of ALDH2 were preserved in only one other ALDH isozyme each; ALDH1B1 has a threonine at the equivalent position T185 and ALDH9A1 has a serine at the equivalent position at S279. It is also interesting to note that in 6 of the remaining 17 ALDHs, the S279 is substituted for with E or D, but none of the equivalent of T185 substitution are negatively charged amino acid mimetics. On the other hand, T412 had much higher conservation in that 12 out of the 18 ALDH isozymes had either a threonine or serine, and 2 members of the ALDH family had a negatively charged amino acid, Asp, at the equivalent position of T412

Finally, in contrast to the low degree of conservation of T185 and S279, we found three other serine/threonine sites that were highly conserved among all the 18 ALDH isozymes: T384 was conserved in 16 of the 18 ALDH isozymes, and T433 and S471 were conserved in all 18 ALDH isozymes. These data suggest that these three sites may be universal serine/threonine phosphorylation sites for the ALDH super gene family. Note that as a reference point for the accuracy of alignment, the critical catalytic site, Cys 302, was found at the equivalent position in 17 of the 18 ALDH isozymes, except for the more divergent member of ALDH16A1.

## Discussion

It is well-established that post-translational modification of proteins and enzyme can modulate the activity of many enzymes, thus playing an important role in cellular functions. Phosphorylation affects the activity of many enzymes through increased interaction with a partner protein [38, 39], inhibition of intramolecular interaction [40, 41], decreased ability to be modified by ubiquitination and subsequent degradation [42, 43] and/or through altered access to the substrate [44, 45]. We previously showed that ALDH2 is a substrate of εPKC and that εPKC-mediated phosphorylation of ALDH2 leads to enhanced catalytic activity towards oxidation of toxic aldehydic substrate and confers cardioprotection against ischemia-reperfusion injury [31]. However, non-enzymatic modification of ALDH2, in particular, on the critical catalytic cysteine 302 residue also occurs by its electrophilic and reactive aldehyde substrate, 4HNE [24]. In our previous studies, we demonstrated that a small molecular agonist of ALDH2, Alda-1, positioned at the substrate tunnel near Cys302 could protect ALDH2 from 4HNE inactivation. Here, we determined whether εPKC phosphorylation or phosphor-memetic of three serine/threonine residues of ALDH2 (T185, S279 and T412) mediate activation of the enzyme and/or protect ALDH2 from 4HNE inactivation.

Mutation of T185 to A did not affect ALDH2 activity (Fig. 2b), and T185E (phosphomimetic mutation) resulted in lower ALDH2 activity relative to wild type or T185A mutant, suggesting a structural role of this residue, and/or that T185 is a site that mediates phosphorylation-induced *inactivation* of ALDH2 (Fig. 2b). T185A and T185E mutants were also sensitive to 4HNE inactivation (Fig. 3), but T185E may have a lower sensitivity, relative to T185A (Fig. 3). Together, these data indicate that although T185 is relatively close to the catalytic site and may protect from 4HNE inactivation when negatively charged, phosphorylation of T185 by εPKC is unlikely to mediate activation of ALDH2. Furthermore, we find that T185 is conserved in species that have εPKC and there is threonine in that position in two species that lack εPKC, further supporting a role of this amino acid in ALDH2 activity. However, its role is not the same for other ALDH isozymes; S or T is not found in any other 18 ALDH isozymes in humans in the equivalent position of T185 (except for ALDH1B1), and only one of the ten species that lack εPKC have the expected phosphomimetic amino acid substitution, which is predicted to make up for the lack of the kinase (Figs. 4 and 5). Together, we conclude that if T185 phosphorylation in ALDH2 is mediated by εPKC, it does not affect ALDH2 catalysis, but it may contribute to protection of ALDH2 from 4HNE-induced inactivation.

Mutation of S279A and T412A each resulted in enzyme with only 50% activity relative to the wild type enzyme (Fig. 2b). Whether the loss of activity reflects a structural defect or role for these two amino acids in catalysis, per se, cannot be determined based on our study. However, whereas mutation to a phosphomimetic E (T412E) resulted in an enzyme with even lower activity relative to T412A, S279E is more active relative to S279A. These data suggest that S279 is the phosphorylation site that mediates the increase in ALDH2 activity by εPKC; indeed, S279E mutant was completely insensitive to further activation by εPKC-mediated phosphorylation (Fig. 2d).

So, what is the role of T412 phosphorylation? T412A is greatly activated by εPKC-mediated phosphorylation (2.5-fold increase in ALDH2 activity relative to non-phosphorylated enzyme; Fig. 2d) and T412A mutant is completely insensitive to 4HNE-induced inhibition of ALDH2 (Fig. 3). We also find that T412 is highly conserved in evolution; even among the species that lack εPKC, 3/10 have S at that position and 5/7 of the remaining species have a phosphomimetic D in that position (Fig. 4, right panel). Finally, in 12 of the 18 other ALDH isoforms in humans, the equivalent of T412 is conserved and 2 of the remaining 6 have a phosphomimetic D at that position. Together, these data suggest an important regulatory role for T412; its phophsorylation may inhibit 4HNE inactivation. Importantly, because the T412E mutant was also less sensitive to εPKC-mediated increase in ALDH2 activity, we conclude that T412 likely also contribute to εPKC-mediated activation of ALDH2. The physical proximity of S279 and T412 in ALDH2 (Fig. 1b) may also contribute to the role of these two putative phosphorylation sites by the same protein kinase, εPKC. We suggest that T412/S279, the two neighboring amino acids on enzyme surface in 3D, are allosteric sites that are protecting ALDH2 from 4HNE inactivation, possibly by altering the structure of the catalytic tunnel and the access of 4HNE to the channel.

The limitations of this in vitro study should be pointed out. Since the first study by Thorsten and Koshland [33] mutation of potential phosphorylation site to an amino acid with a negative charge, to mimic phosphorylation, has been used extensively. Furthermore, mutation of amino acids to an alanine residue seems to be of minimal structural consequences and is therefore often used to identify the role of a particular amino acid; a loss of function is taken to indicate that the particular amino acid is required for that function. However, clearly, any mutagenesis of proteins may have additional ‘gain of function’ consequences due to problem in folding, maturation and/or stability of the enzyme. Furthermore, as all these proteins were expressed in bacteria, they were missing additional co- and post-translational modifications that may affect the activity of the enzyme. Relevant to this point, we found that with one exceptions, all the ALDH2 mutants had lower activity relative to wild type enzyme and that, together with the work with recombinant enzymes remain caveats of our study. Nevertheless, we believe that this work provides the first evidence for the role of particular sites in ALDH2 in responding to εPKC-mediated phosphorylation and to 4HNE-induced inhibition of the enzyme, through a mechanism termed substrate-mediated suicide.

The co-evolution study strengthens our in vitro observations. It was striking to observe that the three ALDH2 phosphorylation sites identified by εPKC appear to co-evolve well with this particular εPKC isozyme. Among all the species that have εPKC, we found that all the three phosphorylation sites were invariably conserved. It implies that there was a strong selection to preserve these three sites for εPKC phosphorylation. It is only in the species where εPKC is absent or lost, these three phosphorylation sites would begin to drift. This co-evolution was even more striking when we aligned all 19 known functional human ALDH isozymes to evaluate the degree of conservation of these putative phosphorylation sites within this supergene family. We found that except for T412 position, which was conserved in 12/18 isozymes, T185 and S279 were unique to the ALDH2 isozyme and one addition isozyme each (ALDH1B1 for T185 and ALDH9A1 for S279). This implies that the co-evolution relationship was uniquely maintained between ALDH2 and εPKC and these three phosphorylation sites may be preferentially regulated by εPKC. We also identified three other serine/threonine residues, T384, T433 and S471 that were extremely well conserved across all the ALDH gene family members. Based on the alignment of 16 known ALDH sequences, Sheikh et al., also identified T384 and S471 as critical conversed amino acids [46]. T384 is located close to the solvent surface and binds to the carbonyl backbone of another conserved amino acid Proline 383. Such interaction appears to be critical for the stability of a local structure in all ALDHs. S471, on the other hand, is located closer to the catalytic tunnel and interacts with residues 269 and 270. Site-directed mutagenesis indicated that mutation at this position would affect the critical conversed general base, Glu268, and dramatically reduced the enzyme activity. Whether these three residues are preserved for ALDH phosphorylation and or for structural effects remain to be determined.

Mitochondrial ALDH2 is a key detoxifying enzyme guarding the integrity and health of this important organelle [27]. As most mammalian cells rely on oxidative respiration for ATP production, mitochondrial lipid bilayer is undoubtedly one of the major cellular sites where lipid peroxidation-derived 4HNE is produced by ROS generated from the electron transport chain [47]. The association between ALDH2, 4HNE accumulation and human disease have been the subject of extensive reviews in recent years [27, 48, 49]. The identification of the sites that mediate εPKC -induced increase in ALDH2 activity to detoxify acetaldehyde, 4HNE and other toxic aldehydes from food, environmental sources and normal metabolism and protection from inactivation by its toxic substrates, such as 4HNE, contributes to our understanding how this mitochondrial enzyme is regulated by signal transduction. We believe that improving mitochondrial health via εPKC activation and its downstream substrate, ALDH2, should be a viable strategy to confer beneficial effects in a variety of human diseases [50]. In the context of human diseases that are associated with ALDH2 activity or ALDH2 mutation, it will therefore be worthwhile to explore in the future the role εPKC-mediated phosphorylation of ALDH2.

## Conclusions

The role of three serine/threonine phosphorylation sites by εPKC on ALDH2 were characterized. Site-directed mutagenesis and in vitro phosphorylation revealed that S279 was a critical εPKC phosphorylation site for the activation of ALDH2. Whereas, phosphorylation of T185, S279 and T412 conferred protection against reactive aldehyde, 4HNE, inactivation of ALDH2. Alignment across a wide range of diverse biological species and of 18 known human ALDH multigene family members showed that the three phosphorylation sites co-evolved tightly with species that expressed εPKC. Such alignment also identified both unique and conserved serine/threonine on ALDH2 and its isozymes. Our findings indicated that εPKC phosphorylation and its coevolution with ALDH2 played an important role in the regulation and protection of ALDH2 enzyme activity.

## Additional files

Additional file 1: Phylogenetic Tree of the 20 species for ALDH2 and εPKC coevolution comparison. Species in green letters are those with a homology of εPKC. Species in red letters are those without a homology of εPKC. (PDF 73 kb)
Additional file 2: The table shows the 10 species with εPKC (left panel), the 10 species without εPKC (right panel) and their amino acid residues at the three human ALDH2 phosphorylation sites, T185, S279 and T412. (PDF 313 kb)

## Abbreviations

4HNE: 4-hydroxy-nonenal
ALDH2: Aldehyde dehydrogenase 2
ALDH: Aldehyde sehydrogenase
C302: Cysteine 302
S279: Serine 279
T185: Threonine 185
T412: Threonine 412
εPKC: Epsilon protein kinase C.
