# Supplementary material for: Aldehyde dehydrogenase 2 activation and coevolution of its εPKC-mediated phosphorylation sites
Source: J Biomed Sci. 2017 Jan 5;24:3. doi: 10.1186/s12929-016-0312-x (PMC5217657; doi:10.1186/s12929-016-0312-x)
Supplement: Additional file 1: — Phylogenetic Tree of the 20 species for ALDH2 and εPKC coevolution comparison. Species in green letters are those with a homology of εPKC. Species in red letters are those without a homology of εPKC. (PDF 73 kb) [file 12929_2016_312_MOESM1_ESM.pdf]

Supplementary Material A

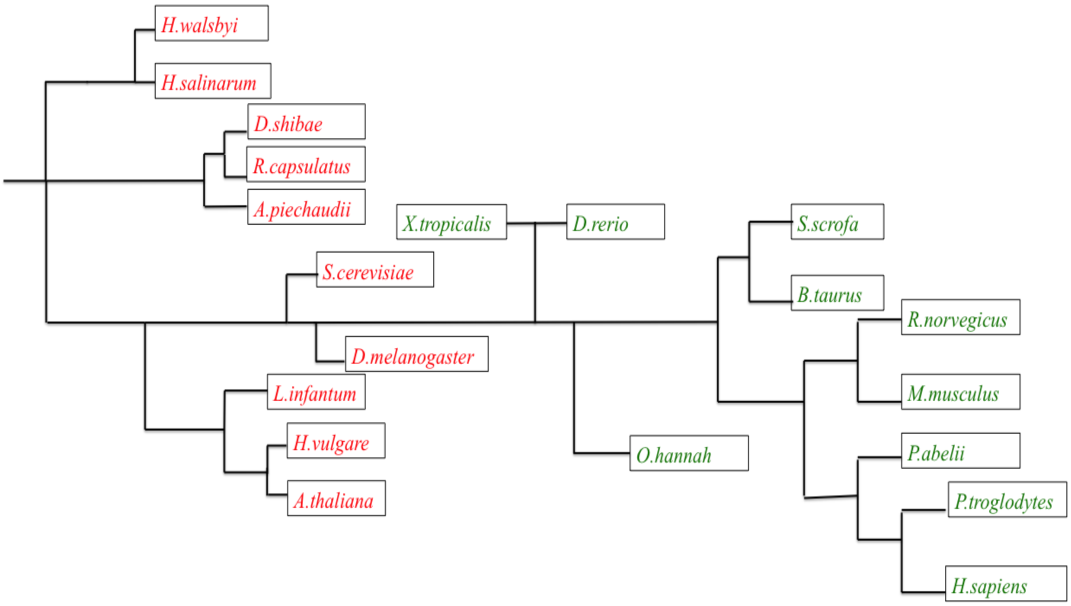

Phylogenetic Tree of the 20 species for ALDH2 and εPKC coevolution comparison. Species in green letters are those with a homology of εPKC. Species in red letters are those without a homology of εPKC
