# Supplementary material for: Aldehyde dehydrogenase 2 activation and coevolution of its εPKC-mediated phosphorylation sites
Source: J Biomed Sci. 2017 Jan 5;24:3. doi: 10.1186/s12929-016-0312-x (PMC5217657; doi:10.1186/s12929-016-0312-x)
Supplement: Additional file 2: — The table shows the 10 species with εPKC (left panel), the 10 species without εPKC (right panel) and their amino acid residues at the three human ALDH2 phosphorylation sites, T185, S279 and T412. (PDF 313 kb) [file 12929_2016_312_MOESM2_ESM.pdf]

Supplementary Material B

**Species with εPKC**	**185**	**279**	**412**	**Species without εPKC**	**185**	**279**	**412**
Homo sapiens	T	S	T	*Drosophila melanogaster*	T	S	K
Pan troglodytes	T	S	S	*accharomyces cerevisiae*	A	I	S
Pongo abelii	T	S	T	*Arabidopsis thaliana*	C	E	D
Mus musculus	T	S	T	*Hordeum vulgare*	C	D	D
Rattus norvegicus	T	S	T	*Leishmania infantum*	L	E	D
Bos taurus	T	S	S	*Achromobacter piechaudii*	A	P	S
Sus scrofa	T	S	T	*Rhodobacter capsulatus*	T	G	G
Ophiophagus Hannah	T	S	T	*Dinoroseobacter shibae*	F	D	S
Danio Rerio	T	S	S	*Halobacterium salinarum*	E	E	D
Xenopus Tropicalis	T	S	T	*Haloquadratum walsbyi*	A	P	D

The table shows the 10 species with εPKC (left panel), the 10 species without εPKC (right panel) and their amino acid residues at the three human ALDH2 phosphorylation sites, T185, S279 and T412.
